# *UMOD* gene mutations in Chinese patients with autosomal dominant tubulointerstitial kidney disease: a pediatric case report and literature review

**DOI:** 10.1186/s12887-019-1522-7

**Published:** 2019-05-08

**Authors:** Jing Yang, Yu Zhang, Jianhua Zhou

**Affiliations:** Department of Pediatrics, Tongji Hospital, Tongji Medical College Huazhong University of Science and Technology, Jiefang Ave. 1095, Wuhan, 430030 China

**Keywords:** Autosomal dominant tubulointerstitial kidney disease, *UMOD* gene mutation, Hyperuricemia, Gout, ESRD

## Abstract

**Background:**

Autosomal dominant tubulointerstitial kidney disease (ADTKD) caused by *UMOD* gene mutation (ADTKD-UMOD) is rare in children, characterized by hyperuricemia, gout, and progressive chronic kidney disease. It usually leads to end-stage renal failure at fiftieth decades. Here, we report a 3-year-old Chinese boy in an ADTKD family caused by a novel *UMOD* gene mutation.

**Case presentation:**

A 3-year-old boy was admitted to our hospital because of persistent hematuria. Urinalysis showed BLD 2+ without proteinuria. The serum levels of uric acid, creatinine and electrolytes were normal. No renal cyst or calculus was found by ultrasonography. Renal biopsy was performed and focal and segmental glomerulosclerosis was found in 4 glomeruli among 35 glomeruli examined. His father was found with end-stage renal disease (ESRD) at the age of 29, and renal ultrasound showed several cysts in both kidneys. A novel heterozygous mutation (c.1648G > A,p.V550I) in exon 8 of *UMOD* gene was identified by whole exome sequencing in the family. SCBC Genome Browser alignment showed that V550 were highly conserved in uromodulin among different species. Software predicted that the mutation is suspected to be harmful. By literature review, there are 12 mutations of *UMOD* gene in 14 Chinese families including only one pediatric case(a 16-year-old girl).

**Conclusions:**

A novel heterozygous mutation (c.1648G > A,p.V550I) in exon 8 of *UMOD* gene was found in in a Chinese child case with ADTKD-UMOD, which extends our understanding of *UMOD* gene mutation spectrum and phenotype of ADTKD-UMOD in children.

## Background

Autosomal dominant tubulointerstitial kidney disease caused by *UMOD* gene mutation (ADTKD-UMOD) was proposed by KDIGO in 2015 [1].It was previously known as familial juvenile hyperuricemic nephropathy (FJHN), medullary cystic kidney disease type 2(MCKD 2) and UMOD-associated kidney disease [2]. ADTKD-UMOD is a rare disease, almost all patients present the typical manifestation during adulthood, thus very few pediatric cases could be diagnosed in the early years of their life. Up till now, no more than 2000 families have been reported worldwide [3]. The main clinical manifestations of ADTKD-UMOD include hyperuricemia and gout, some patients have mild urinary abnormalities. It usually develops to end-stage renal disease (ESRD) at 30–60 years old. Jonathan et al. reported that the mean age of progression to ESRD was at 56 years old [4], however, there are no Chinese patients included in the study. Histologically, ADTKD-UMOD is characterized by diffuse tubulointerstitial fibrosis with moderate inflammatory cell infiltrate and tubular atrophy. Renal cysts are not always detected, mainly at the corticomedullary junction [5]. As for the treatment of the disease, there is no specific therapy of the disease. Regular dialysis or kidney transplantation is required when the patients have developed to end-stage renal disease.

Here, we report a ADTKD family with *UMOD* gene mutation and summarized the clinical features and types of Chinese patients with *UMOD* gene mutation by literature review.

## Case presentation

**Fig. 1:**
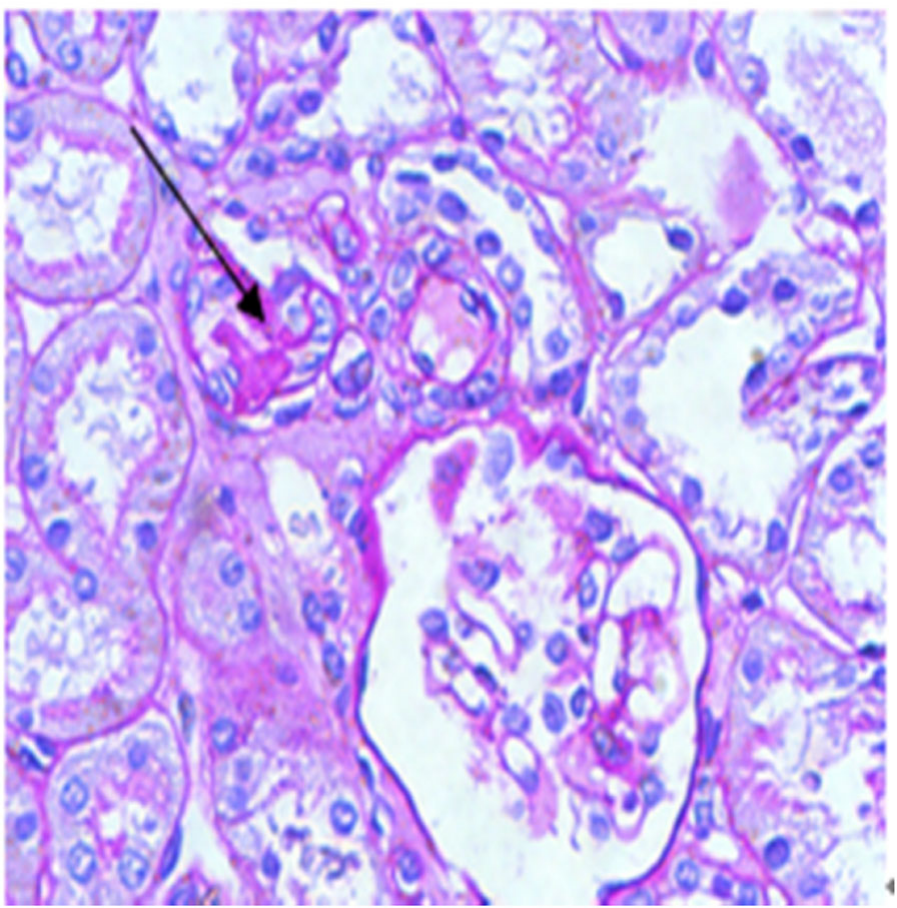
Light micrograph of two glomeruli from the proband. Arrow indicates a small atrophy glomulus with segmental sclerosis

**Fig. 2:**
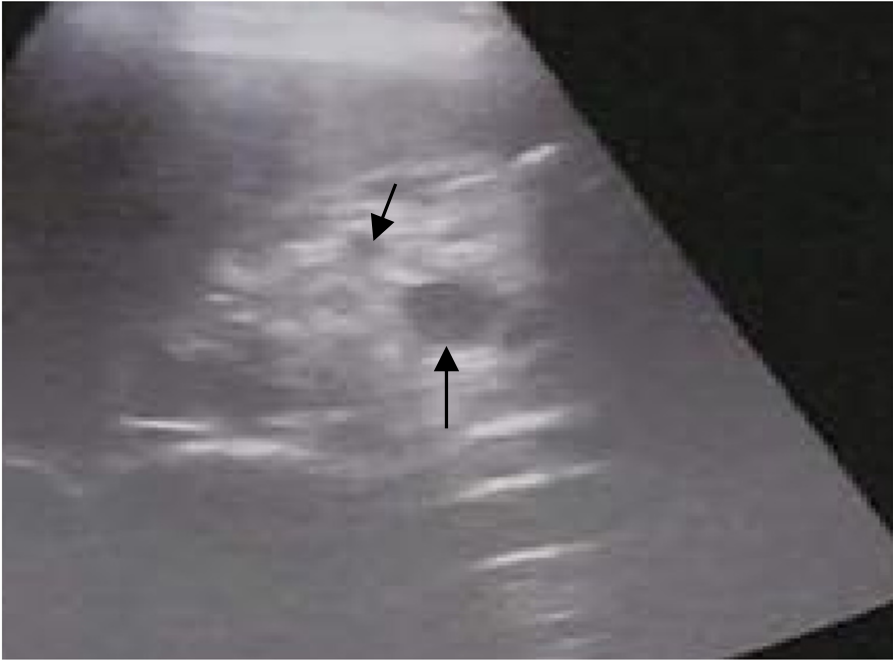
Renal ultrosonograph of the proband’s father. The hyperechogenicity and cysts of different sizes are shown with arrows

**Fig. 3:**
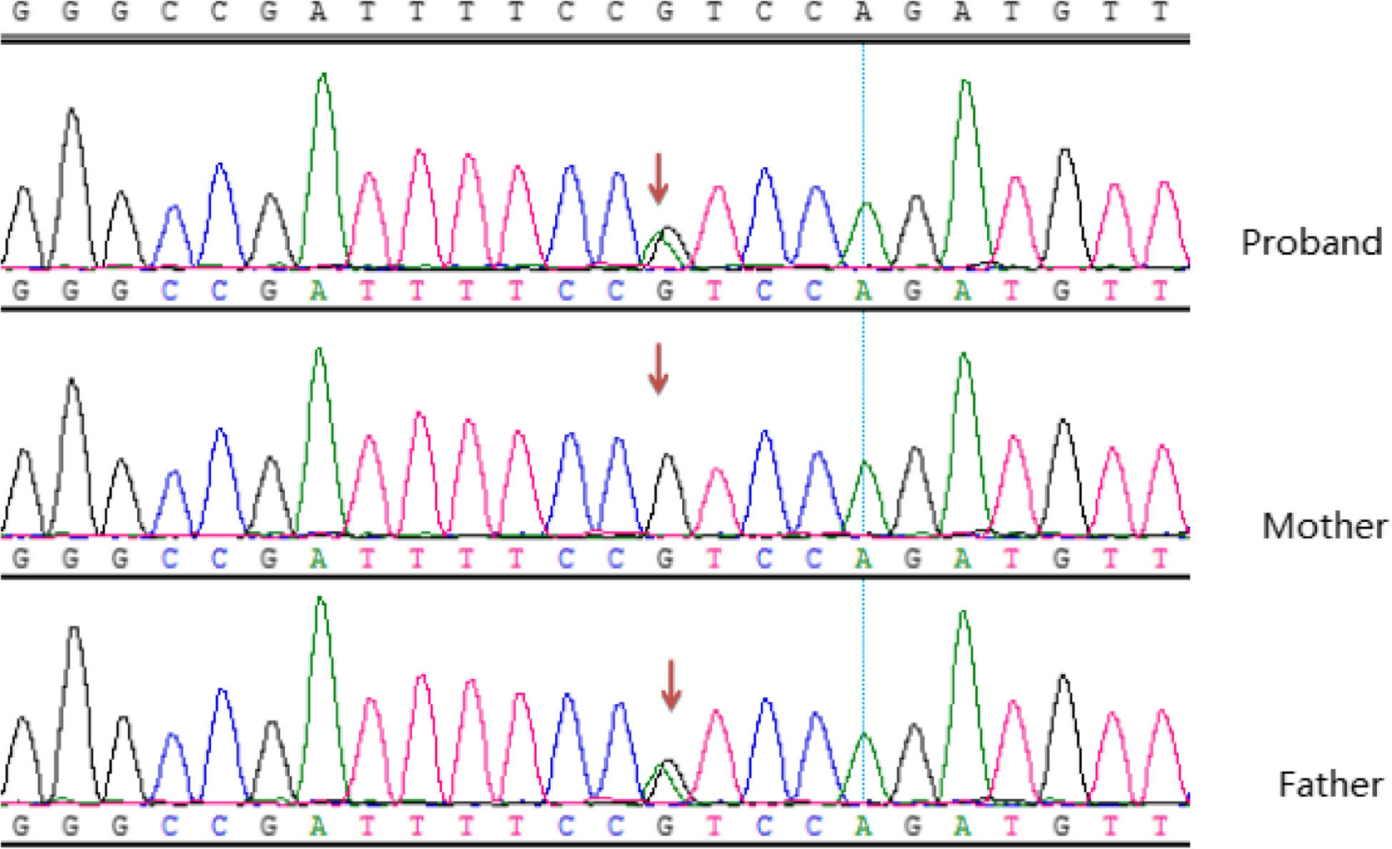
Sanger sequencing of the proband and his patients. The proband and his father are heterozygous mutation, the site of missense mutation (c.1648G > A, p.V550I) is shown with an arrow

**Fig. 4:**
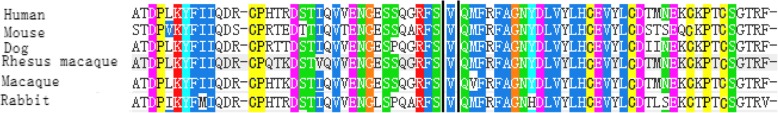
Protein sequence alignment of the V550I mutation regions among different species. The mutated valine is highly conserved among different species and highlighted in black frame

The index patient was a 3-year-old boy, and he was admitted to our hospital with repeated microscopic hematuria. Physical examination revealed no significant abnormality. The proband’s urine analysis just showed occult blood 2+ and no proteinuria. His serum creatinine level was 27 umol/L, and uric acid level was 175 umol/L. Serum IgG, IgA, IgM, C3 and C4 levels were normal, and ANA, dsDNA, and ANCA were negative. No cyst and high echogenicity were found in renal ultrasonography. Renal biopsy showed 4/35 glomerular segmental sclerosis, immunofluorescence were negative, renal interstitial fibrosis and renal tubular atrophy (Fig. 1). His father was found with end-stage renal disease (ESRD) (Scr 1400umol/L) at the age of 29, and hematuria, proteinuria, edema and hyperuricemia (UA 776umol/L). Renal ultrasound showed several cysts in both kidneys (Fig. 2). Other family members have no clinical manifestation of gout, CKD. There is a novel missense mutation(c.1648G > A,p.V550I) in exon 8 of *UMOD* gene, resulting in the conversion of valine to isoleucine. This mutation is extremely rare in the population, merely 0.0003 in the dbSNP database and 0.0009 in the Hapmap database for Asians. At present, there is no literature report on the pathogenicity of c.1648G > A mutation in the *UMOD* gene. This resulted in amino acid change that may affect the normal function of the protein. Sanger sequencing showed that the father and the pediatric patient carried the same mutation (Fig. 3), in addition, the father’s clinical phenotype was consistent with ADTKD-UMOD. SCBC Genome Browser alignment results indicated that V550 in *UMOD* gene was highly conserved among different species (Fig. 4) and its mutation to isoleucine is predicted to be harmful by Software analysis.

## Literature review of Chinese patients with *UMOD* gene mutation

To date, more than 100 mutations have been reported in *UMOD* gene in the world. Most of them are missense mutations located in the N-terminus of the protein (95% are located in exon 3 and 4 of *UMOD* gene) [6]. There are 12 different mutations of *UMOD* gene in 14 Chinese families with ADTKD (Table 1) [7–12]. Among the 14 patients, 9 patients had higher UA level, 7 patients had kidney cysts, 13 patients had a positive family history. As described in KDIGO consensus in 2015, positive family history is a very important clue to the diagnosis of ADTKD-UMOD [1]. The patients developed to ESRD at different ages (varied from 16 years old to 60 years old). Among the 12 mutations of *UMOD* gene, 8 mutations were found in exon 3, 2 in exon 4, 1 in exon 5, and 1 in exon 9. Now we found a novel mutation (c.1648G > A,p.V550I) in exon 8 of *UMOD* gene, which was not reported in previous Chinese cases. We believe this is the first mutation in exon 8 of *UMOD* gene in Chinese patients.

**Table 1:** Summary of the Chinese patients with *UMOD* mutation

Index case	Year	Sex	Age (yr)	UA	Cyst	ESRD	Family History	exon	Mutation
1	2018	F	16	7	No	No	Yes	3	c.667 T > G,p.Cys223Gly
2	2013	M	18	8.2	Yes	Yes	Yes	3	c,707G > A,p.Pro236Gln
3	2017	M	20	8.4	No	No	No	3	c.104G > A,p.Cys35Tyr
4	2013	F	21	10.1	Yes	No	Yes	3	c.744C > G,p.Cys248Trp
5	2015	F	21	4.8	Yes	No	Yes	3	c.744C > G,p.Cys248Trp
6	2015	M	22	8.8	No	Yes	Yes	3	c.1153C > T,p.Arg385Try
7	2013	F	24	10.1	No	Yes	Yes	3	c.326 T > A,p.Val109Glu
8	2015	F	24	6.8	No	No	Yes	3	c.707G > A,p.Pro236Gln
9	2017	F	38	2.8	No	No	Yes	3	c.113A > T,p.Asn38Ile
10	2017	F	41	5.4	Yes	No	Yes	4	c,860G > T,p.Cys28Phe
11	2015	M	44	4.3	Yes	No	Yes	5	c.197 T > C,p.Leu66Pro
12	2012	M	45	9.6	No	Yes	Yes	9	c.1815A > G,p.Thr605Gly
13	2015	M	47	9.0	Yes	Yes	Yes	4	c.854C > A,p.Ala285Glu
14	2015	M	60	4.7	Yes	No	Yes	3	272delC

*M* male, *F* female, *UA* uric acid(mg/dl)

## Discussion and conclusions

The present paper has reported a novel missense mutation (c.1648G > A) in exon 8 of *UMOD* gene in a Chinese family with ADTKD, resulting in the conversion of valine to isoleucine (p.V550I) in uromodulin. This is the first reported mutation in exon 8 of *UMOD* gene in the Chinese population. Software predicts this mutation is suspected to be harmful. Combined with the clinical features, family history and genetic testing results of the children, we consider that this is an ADTKD-UMOD family. At present, the child has no typical clinical manifestations and only showed mild hematuria due to the younger age. His father developed to ESRD at 29-years and received a kidney transplant.

*UMOD* gene is located on chromosome 16p12.3-p13.11, including 11 exons, of which exons 2–11 are the coding region for uromodulin [7]. Uromodulin, also known as Tamm-Horsfall glycoprotein, is the most abundant protein secreted in normal urine. It is exclusively produced by tubular cells in TAL, contains three epidermal growth factor (EGF)-like domains, an eight-cysteine-containing-domain in a cysteine-rich region and a zona pellucide domain that is responsible for the polymerization of extracellular protein into helical filaments [13, 14].

Here, the novel mutation (c.1648G > A, p.V550I) we reported is located in exon 8, the ZP domain of uromodulin. The uromodulin is polymerized through its ZP domain, which is a common conserved module in many extracelluar eukaryotic protein that can assemble into matrices [15].

Although the role of uromodulin is still unclear, studies on *UMOD* knockout mouse suggested it may play a role in regulating the water/electrolyte balances [5]. Renigunta et al. showed that uromodulin expression positively regulates its transmission to the plasma membrane through direct interactions, thereby significantly increasing the activity of the ROMK2 potassium channel [16]. Uromodulin also promotes the transport of the furosemide-sensitive Na + −K + -2Cl-cotransporter NKCC2, which phosphorylation increases NKCC2 activity and Na + absorption. Therefore, dysfunction or insufficient secretion of uromodulin can lead to decreased Na + reabsorption and increased excretion [6]. Uromodulin plays an important role in preventing urinary tract infections. A study by Bates et al. showed that the mice deficient of Tamm-Horsfall protein were more susceptible to urinary tract infection [17]. Uromodulin also plays an important role in the prevention of renal stone. Studies have shown that the Tamm-Horsfall protein has an antioxidant effect that protects renal tubular cell from free oxygen radical damage. Since the damage of the cells promotes the retention of calcium oxalate crystal on the cell membrane, it promotes the growth of stone. Because of its antioxidant capacity, the Tamm-Horsfall protein may be an inhibitor of calcium oxalate stone formation [18]. It has been suggested that uromodulin plays a role in the innate immunity of the kidney [5].

The pathogenesis of uromodulin-associated kidney diseases is not fully understood, Bernascone et al.speculated that mutant uromodulin aggregated in ER, could cause epithelial cell damages, interstitial inflammation and fibrosis. The formation of renal cysts could be a consequence of progressive TAL cellular damage and secondary proliferation [19].

Trudu et al. [20] proposed that TAL stress and inflammatory signals represent an early event in ADTKD-UMOD. And their results also suggested that renal damage occurred in the distal tubules first, where uromodulin is expressed and is then spread to neighbouring proximal tubules.

The novel heterozygous mutation (c.1648G > A,p.V550I) we reported here is the first reported mutation in exon 8 of *UMOD* gene in Chinese patients. This mutation is located in ZP domain of uromodulin, which is responsible for the polymerization of extracellular protein into helical filaments. The mutant uromodulin can accumulate in the ER, which can lead to TAL structural injury. In addition, inflammatory response resulted in a progressive interstitial fibrosis and tissue scarring. Finally, it can lead to renal failure.

Up to now, there are more than 100 mutations of *UMOD* gene in ADTKD-UMOD. Most of them were found to be point mutation resulting in substitution of cysteine in exon 3 and 4 of *UMOD* gene [6]. Among Chinese patients, 12 mutations were found in exon 3,4,5,9 of *UMOD* gene, most of them in exon 3, rare in exon 4. This seems a little bit different to that in USA and Europe, and needs more ADTKD-UMOD case accumulation.

There are still some shortcoming in our study. In the future, we need to perform functional verification to determine the role of the mutation and its effects on protein.

In summary, a novel mutation (c.1648G > A,p.V550I) was found in exon 8 of *UMOD* gene in a Chinese child case with ADTKD-UMOD, which extends our understanding of *UMOD* gene mutation spectrum and phenotype of ADTKD-UMOD in children. The clinical manifestations of ADTKD-UMOD are not typical in children, it may only present with mild hematuria, therefore a positive family history is a key clue for the diagnosis of such disease in children.

## Abbreviations

ADTKD: autosomal dominant tubulointerstitial kidney disease
ADTKD-UMOD: autosomal dominant tubulointerstitial kidney disease caused by *UMOD* gene mutation
ANA: antinuclear antibody
ANCA: anti-neutrophil cytoplasmic antibody
D8C: domain of eight cysteines
dsDNA: double strands DNA
EGF: epidermal growth factor
ESRD: end-stage renal disease
UA: uric acid
UAKD: uromodulin-associated kidney disease
ZP: zona pellucide
